# Anti-Phospholipid Antibodies and COVID-19 Thrombosis: A Co-Star, Not a Supporting Actor

**DOI:** 10.3390/biomedicines9080899

**Published:** 2021-07-27

**Authors:** Francisco Javier Gil-Etayo, Sara Garcinuño, Antonio Lalueza, Raquel Díaz-Simón, Ana García-Reyne, Daniel Enrique Pleguezuelo, Oscar Cabrera-Marante, Edgard Alfonso Rodriguez-Frias, Alfredo Perez-Rivilla, Manuel Serrano, Antonio Serrano

**Affiliations:** 1 Department of Immunology, Hospital Universitario 12 de Octubre, 28041 Madrid, Spain; fgile@salud.madrid.org (F.J.G.-E.); dpleguezuelo@salud.madrid.org (D.E.P.); oscar.cabrera@salud.madrid.org (O.C.-M.); edgardalfonso.rodriguezde@salud.madrid.org (E.A.R.-F.); 2 Instituto de Investigación Sanitaria Hospital 12 de Octubre (imas12), 28041 Madrid, Spain; garcinunosara@gmail.com; 3 Department of Internal Medicine, Hospital Universitario 12 de Octubre, 28041 Madrid, Spain; antonio.lalueza@salud.madrid.org (A.L.); rdiazs@salud.madrid.org (R.D.-S.); agreyne@salud.madrid.org (A.G.-R.); 4 Department of Microbiology, Hospital Universitario 12 de Octubre, 28041 Madrid, Spain; alfredo.perez@salud.madrid.org; 5 Department of Immunology, Hospital Universitario Clínico San Carlos, 28041 Madrid, Spain; mserranobl@gmail.com; 6 Department of Epidemiology, Biomedical Research Centre Network for Epidemiology and Public Health (CIBERESP), 28029 Madrid, Spain

**Keywords:** COVID-19, thrombosis, antiphospholipid syndrome, antiphospholipid antibodies, autoimmunity

## Abstract

Background: COVID-19 clinical features include a hypercoagulable state that resembles the antiphospholipid syndrome (APS), a disease characterized by thrombosis and presence of antiphospholipid antibodies (aPL). The relationship between aPL-presence and the appearance of thrombi as well as the transience or permanence of aPL in COVID-19 patients is not sufficiently clear. Methods: A group of 360 COVID-19 patients were followed-up for 6 months. Classic aPL, anti-B2GPI IgA, anti-phosphatidylserine/prothrombin IgG/M and anti-SARS-CoV-2 antibodies were determined at acute phase and >12 weeks later. The reference group included 143 healthy volunteers of the same age-range distribution. Results: aPL prevalence was similar in COVID-19 patients and the reference population. aPL presence in both determinations was significantly associated with thrombosis (OR: 2.33 and 3.71), strong agreement being found for classic aPL and anti-B2GPI IgA (Weighted kappa: 0.85–0.91). Thrombosis-associated aPL occurred a median of 17 days after hospital admission (IQR: 6–28) vs. 4 days for the rest (IQR: 3–7). Although anti-SARS-CoV-2 antibodies levels increased during convalescence, aPL hardly changed. Conclusions: Most COVID-19 patients would carry these aPL before the infection. At least two mechanisms could be behind thrombosis, early immune-dysregulation-mediated thrombosis after infection and belated-aPL-mediated thrombosis, with SARS-CoV-2 behaving as a second hit.

## 1. Introduction

COVID-19 is a pathology characterized by a wide spectrum of clinical profiles [1]. COVID-19 has been strongly associated with a hypercoagulable state, especially with thrombotic events [2,3]. These coagulation abnormalities include increased D-Dimer, prolongation of prothrombin time and thrombocytopenia [4], which are highly related to an unfavorable outcome [2,5].

The COVID-19 hypercoagulable state appears to be a mixture of other prothrombotic situations [6,7]. Among these situations, the antiphospholipid syndrome (APS) is the one that most closely resembles what happens in COVID-19 [8,9], which has led to the study of the presence of antiphospholipid antibodies (aPL) in these patients [10,11,12].

Classification criteria for thrombotic APS require the presence of at least one laboratory criterion and one clinical criterion (arterial, venous, or small vessel thrombosis). Laboratory criteria include the following: lupus anticoagulant (LA), anti-cardiolipin (anti-CL) or anti-B2-Glycoprotein-I (anti-B2GPI) of IgG/IgM isotypes in two consecutive determinations distanced 12 weeks apart [13]. Recently, additional aPL associated with thrombotic events but not listed in the classification criteria (extra-criteria aPL) have been described. Among the extra-criteria aPL, the most commonly detected in association with thrombosis are IgA isotype of anti-B2GPI and IgG/IgM isotype of anti-phosphatidylserine/prothrombin (anti-PS/PT) [14,15,16]. Likewise, there are also other clinical manifestations of APS that are not included in the classification criteria (extra-criteria manifestations) [17,18].

The prevalence of aPL in COVID-19 patients, with or without thrombotic events, varies between 21–70%, according to the methods used in its detection and whether the presence of LA is assessed or not [19,20,21]. In a recent meta-analysis that included 21 studies, the pooled prevalence rate of one or more aPL (aCL or anti-B2GPI IgG/M/A, or anti-PS/PT, or LA) was 46.8% [22]. In our country, the prevalence of aPL in COVID-19 patients at the time of hospital admission, excluding LA (aCL or anti-B2GPI, or anti PS/PT), is 23,6% which is higher than the 6.1% described in the control group of 201 blood donors [21] and the 5–5.5% described in other studies [23,24]. LA is the most frequent aPL associated to COVID-19, but its presence is transitory [25] and has not been associated with an increased risk of thrombosis [26].

It is well known that aPL can appear in the context of various infections [27,28]. To avoid these false positives the APS classification criteria establish the condition of persistent positivity of aPL: a positive test must be confirmed by a second positivity a minimum of 12 weeks after the first evaluation [13].

It has been reported that some infections like hepatitis C, herpes zoster and Q fever may be involved in triggering APS events, including the APS catastrophic form [29,30,31]. The same association has been reported in SARS-CoV-2 infection, which could induce the synthesis of criteria and extra-criteria aPL (mainly IgA anti-B2GPI) [12,20]. However, although it has been observed that the positivity of LA is transitory [25], the persistence beyond 12 weeks of the classic aPL and especially of the extra-criteria aPL, in large series of patients with COVID-19 has not been sufficiently addressed.

Zhang et al. reported three patients with stroke with positive anti-CL IgA and anti-B2GPI IgG and IgA [12]. Harzallah and collaborators described 25 patients with positive LAC and five with anti-CL or antiB2GPI IgG or IgM in a cohort of 56 COVID-19 patients [32]. Devresse et al. reported 23 of 31 patients positive for at least one criteria aPL. Repeat testing did not confirm the positivity of those aPL in all patients one month after the infection [33].

This work has aimed to evaluate the variability in the levels of aPL at the time of diagnosis of COVID-19 and at least 180 days after the first sample and the association of those aPL with COVID-19 complications in a prospective series of 360 patients who recovered from the infection. aPL dynamics were compared to the evolution of anti-SARS-CoV-2 antibodies.

## 2. Materials and Methods

### 2.1. Study Design

We performed a prospective observational study including COVID-19 patients followed-up for at least 180 days from hospital admission. Classic and extra-criteria aPL serum levels were determined at admission and in a second sample obtained at least 12 weeks later.

### 2.2. Patients

Patients were enrolled in the “Hospital Universitario 12 de Octubre” from March to October 2020. Inclusion criteria were: (1) Patients over 18 years with symptoms of acute COVID-19 infection; (2) Clinical characteristics that required hospital admission; (3) Confirmed diagnosis by RT-PCR; (4) Clinical follow-up for at least 6 months; (5) Quantification of aPL in a sample taken on the first day after hospital admission and in a second sample taken at least 12 weeks later.

A total of 201 out of the 561 patients selected initially were excluded either because a second serum sample was not available or because clinical follow-up was interrupted). Finally, 360 COVID-19 patients (72% Caucasian, 24% born in South and Central America and 2% of other origins) were enrolled in this study. Age: mean 58.1 years and median 59 years (IQR: 46.5–69). The algorithm of disposition and outcomes is described in Supplementary Figure S1.

### 2.3. Reference Population

A reference group formed by 143 healthy individuals with a representative age-range of the real age-distribution in general population of the same geographic area was selected to compare COVID-19 patients with a healthy population of the same area. Age: Mean 61.3 years; median 64 years (IQR: 50–75).

Blood donors generally constitute an excellent reference population of healthy people; however, this entails a bias of people over 50 years and those over 65 years are not included, so the most common age range in COVID-19 patients in our environment (over 50 years) are underrepresented. The reference group consisted of 33 blood donors and 110 volunteers recruited from people over 40 years who underwent a preoperative study for minor conditions not related to any major disease (such as ophthalmic cataract surgery). The selected patients had no history of serious systemic or vascular pathologies and no symptoms other than minor age-related symptoms at the time of the medical examination.

### 2.4. Study Definitions

COVID-19 case was defined as a positive result for SARS-CoV-2 according to reverse transcription polymerase chain reaction (RT-PCR) assay performed on nasal swab sampling from patients with COVID-19 consistent symptoms.

Ventilatory failure: was defined as a PaO2/FiO2 < 300 (blood oxygen pressure/fractional inspired oxygen), or the need for mechanical ventilation (either non-invasive positive pressure ventilation or invasive mechanical ventilation).

Classic aPL: The aPL included in the Sidney’ APS classification criteria [34] excluding LA: anti-CL or anti-B2GPI of IgG/IgM isotypes.

Extra-criteria aPL: aPL not included in the APS classification criteria [34]: IgA aβ2GPI and aPS/PT antibodies of isotypes IgG/IgM.

### 2.5. Samples

Sera from coagulated whole blood samples were collected in the first 24 h after hospitalization (First sample) and at least 12 weeks later (Second sample).

### 2.6. Criteria aPL Determination

IgG and IgM isotypes of anti-B2GPI and anti-CL were determined by addressable laser bead immunoassay (ALBIA) using BioPLex 2200 multiplex immunoassay system APLS (Bio-Rad, Hercules CA, USA). The cutoff to consider IgG and IgM anti-B2GPI and anti-CL as positive was 18 U/mL, according to the 99th percentile evaluated in a healthy population. LA was measured in a sample of 67 patients using HemosiL dRVVT (cutoff ratio 1.2) and HemosiL Silica Clotting Time (cut-off ratio 1.3) assays (Instrumentation Laboratory SpA, Milano, Italy).

### 2.7. Extra-Criteria aPL Determination

IgA anti-B2GPI were quantified using the QUANTA Lite B2 GPI IgA (INOVA Diagnostics Inc., San Diego, CA, USA), due to the low sensitivity of bead-based immunoassays for these antibodies [35]. Anti-PS/PT of IgG and IgM isotypes were evaluated by enzyme-linked immunosorbent assay (ELISA) using QUANTA Lite anti-PS/PT (INOVA DIAGNOSTICS, San Diego, CA, USA). Cutoffs used to consider the results as positive were: >20 U/mL for IgA anti-B2GPI, >30 U/mL for IgG anti-PS/PT and >40 U/mL for IgM anti-PS/PT. These values correspond to the 99th percentile of a healthy population [18].

ELISA procedures were performed in a Triturus ^®^ Analyzer (Diagnostics Grifols, S.A. Barcelona, Spain).

### 2.8. Anti-SARS-CoV-2 Antibodies Determination

IgG antibodies against the receptor-binding domain (RBD), spike 1 (S1), spike 2 (S2), and nucleocapsid (NCap) structural proteins of SARS-CoV-2 were quantified in paired samples of 126 COVID-19 patients using ALBIA technology with the BioPlex^®^2200 SARS-CoV-2 IgG Panel (Bio-Rad, Hercules CA, USA). The manufacturer’s recommended cutoff was used. Samples showing antibody levels above the detection limit of the system were normalized to the detection limit value.

### 2.9. Data Collection

Clinical data including vascular and thrombotic events, Intensive Care Unit (ICU) admission and ventilatory failure were collected from the electronic medical records and integrated into an anonymized database. Biological and immunological markers were also included.

### 2.10. Statistical Analysis

Results of discrete variables were expressed as absolute frequency and percentage. Association between qualitative variables was determined with Pearson’s chi-square test or Fisher’s exact test, when appropriate. The relative measure of an effect between subgroups of patients was expressed as relative risk based on a 95% CI when considering the aPL positivity as the exposure.

Results of the continuous variables were expressed as median accompanied by the interquartile range (in parentheses) and mean with standard deviation. Mann–Whitney U test was used for comparisons. The non-parametric Wilcoxon Signed-Rank test was used to compare continuous variables (paired data) that were not normally distributed. Inter-rater reliability for qualitative variables was measured using weighted Cohen’s kappa (κ); strength of agreement was interpreted using a rule of five described previously [36].

Multivariate analyses were performed through logistic regression model using variables that presented a *p*-value < 0.11 in a previous univariate analysis. The relative measure of an effect was expressed as odds ratio.

Probabilities under 0.05 were considered significant. Data were analyzed with MedCalc for Windows version 19.3 (MedCalc Software, Ostend, Belgium).

### 2.11. Ethical Issues

This study was conducted in accordance with the principles of the Declaration of Helsinki and was approved by the Clinical Research Ethics Committee of Hospital Universitario 12 de Octubre (reference numbers 20/117, 18/182 and 18/009. Oral or written informed consent was obtained from all patients and members of the reference group.

## 3. Results

### 3.1. aPL Prevalence in COVID-19 Patients

Median age of the 360 COVID-19 patients was 59 years (IQR: 46.5–69) with a discrete smaller proportion of women (41%), however no significant differences were observed in ages between men and women: 59 (IQR: 47–68.3) vs. 59 (IQR: 46–70.8), *p* = 0.937. Median age in the reference population was 64 years (IQR: 51–75). The mean separation time between the two serum samples was 21 weeks (SD: 6.2) and the median was 19.9 (IQR: 16.4–24.3).

We found 63 (17.5%) patients positive for the aPL in the first blood sample collected during the acute phase of the disease. We observed that 16 (4.4%) of the patients presented as least one classic aPL, whereas 47 (13.2%) patients were positive for at least one extra-criteria aPL, 40 patients (11.1%) for anti-B2GPI of IgA isotype and 15 (4.2%) for anti-PS/PT (any isotype). A total of 85% of IgA anti-B2GPI positive patients (*n* = 34) were negative for the other aPL (Isolated positives). The number and type of aPL positivity in the first and second samples can be seen in Supplementary Figure S2 (A, first sample. B, second sample). No significant differences were observed in the prevalence of aPL in the reference population compared to COVID-19 patients, both in the first and in the second sample (Table 1). LA was evaluated in 67 patients, 13 (19%) were positive. The Supplementary Table S1 shows the prevalence of aPL in a group of 320 anonymous blood donors, comparing it with that observed in the reference population and in COVID-19 patients.

Median D-dimer levels in COVID19 patients was 715 ng/mL (IQR: 434–1349), 70% of patient had D-dimer above the cutoff (500 ng/mL). No significant differences (*p* = 0.663) were observed D-dimer levels in aPL positive (758 ng/mL, IQR: 484–1579) versus aPL negative (696 ng/mL, IQR: 430–1323). Patients with thrombotic events, had higher levels (4082.5 ng/mL, IQR: 1579.8–7004.6) than those without thrombi (665 ng/mL, IQR: 586.9–751.2, *p* < 0.001).

### 3.2. Variability of Antibodies over the Time in COVID-19 Patients

On analyzing the aPL levels in paired samples we observed that the Hodges–Lehman median difference between first and second samples for all studied aPL was discrete, with all below 1 U/mL (Table 2). The maximum median difference was 0.11 times the value of the median in the first sample (median difference Index). However, when the levels of antibodies against RBD, S1, S2 and Ncap antigens of SARS-CoV-2 were evaluated, a significant increase in the titer was observed against all of them (Table 2), with median difference index between 2.82 and 7.33.

**Figure 1:**
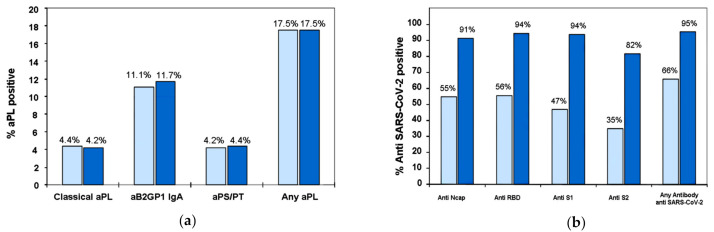
Prevalence of antibodies in first (Light blue) and second serum samples (dark blue). (**a**). Antiphospholipid antibodies. (**b**). Anti-SARS-CoV-2 antibodies. Classic aPL: Anti-Cardiolipin or anti-B2GPI (aB2GPI) of isotypes IgG/M. Anti-B2GPI IgA (aB2GPI IgA), anti-PS/PT (aPS/PT): Anti-phosphatidylserine/prothrombin antibodies of IgG/M isotypes.

No significant differences were observed in the proportion of positive patients for aPL between the first and second samples (Figure 1a). In the analysis of the agreement of aPL in the first and second samples, we found a strong consensus for the classic aPL and IgA anti-B2GPI (Weighted kappa 0.85–0.91) (Table 3). Patients who were negative in the first sample and positive in the second sample were located in a grey area very close to the cutoff. Concordance between the measurements of anti-PS/PT was weak (Weighted kappa 0.43–0.52).

The high concordance in the aPL contrasts with the increase levels of anti-SARS-CoV-2 IgG antibodies in the second sample (Figure 1b).

### 3.3. Clinical Evolution of COVID-19 Patients

During the follow-up, 97 patients (27%) had ventilatory failure and 36 (10%) patients required admission to the ICU, however, no association was found between these outcomes and the presence of aPL (Supplementary Table S2). A total of 37 patients (10.3%) suffered thrombotic events (TE). No association was observed between clinical outputs, ethnic origin or the presence of antibodies against specific antigens of the virus (not shown). Only one of the 37 patients with thrombosis had systemic lupus erythematosus (SLE).

**Figure 2:**
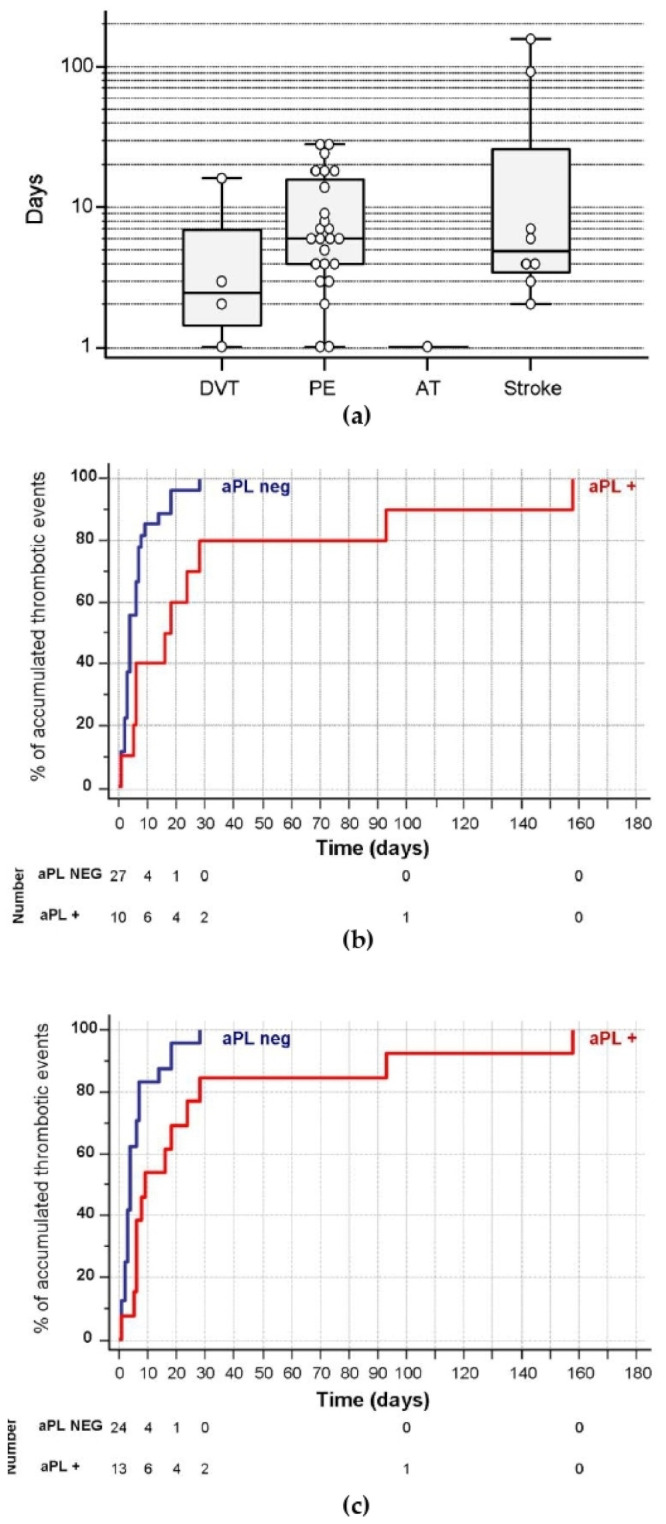
Days of appearance of thrombotic events counted from the day of hospital admission. (**a**). Depending on the type of event. (**b**). Time to onset of thrombotic events in patients who were positive (red) and negative (blue). (**c**). Time to onset of thrombotic events depending on aPL positivity in second sample. Red: aPL positive patients. The number at risk on the different days is indicated in the lower lines. DVT: Deep venous thrombosis. PE: Pulmonary embolism AT: Arterial thrombosis.

The most common TE was pulmonary embolism (PE) with 24 patients, followed by eight who had had thrombotic stroke, and four patients who had deep vein thrombosis (DVT and 1 arterial thrombosis). Two PE patients also had DVT (they were classified in the subgroup of PE). Median time of onset of the first TE with respect to the patient’s admission was 6 days (IQR: 3–12.8) and mean time was 12.5 days. Figure 2a shows the time of appearance of the first thrombotic event according to the type of thrombosis. The thrombotic events that occurred in patients admitted to the ICU were later (median 14 days, IQR: 5.8–25 than in the rest of the patients (median 5.5 days, IQR: 3.0–8.5), although these differences were not significant (*p* = 0.174).

Patients with TE were younger than the rest of the patients. No significant differences were observed in the sex ratio, cardiovascular risk factors or ICU requirement (Table 4A). 

In the acute phase (first sample), aPL positivity was not associated with the development of TE (Table 4A), ventilatory failure or ICU admission (Supplementary Table S2). However, if only classic aPL are considered, the incidence of thrombotic events in aPL positive patients is significantly higher compared to aPL negative patients (RR: 3.36, 95%CI: 1.51–7.46; Table 4A). There was no significant association of LA with thrombosis, ventilatory failure or admission to the ICU.

**Figure 3:**
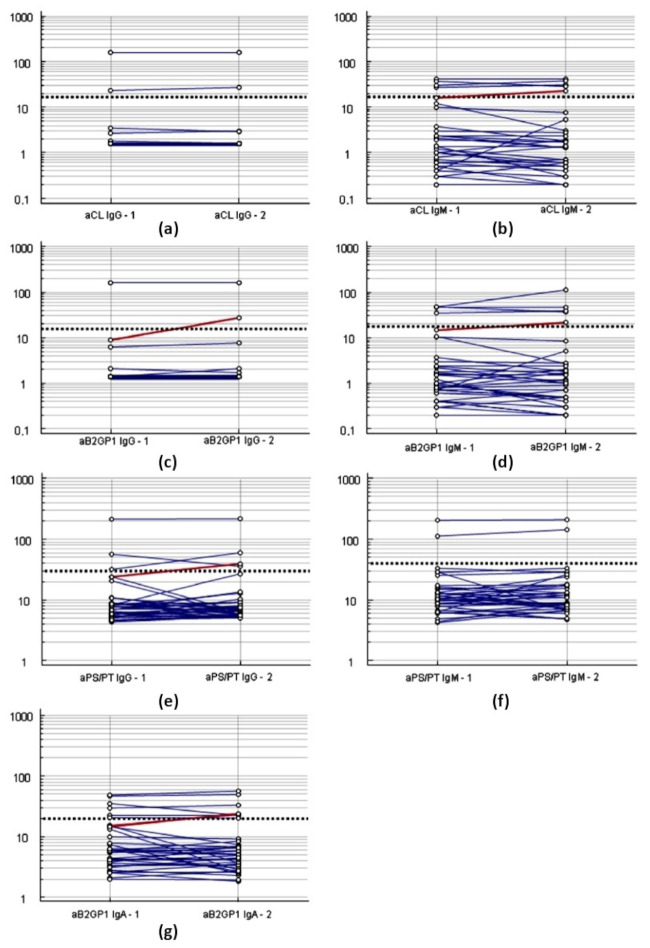
Dot and line diagram representing the Wilcoxon test of the paired samples of antiphospholipid antibodies in COVID-19 patients with thrombotic events. (**a**). Anti-cardiolipin (aCL) IgG. (**b**). aCL IgM. (**c**). Anti-B2GPI (aB2GPI) IgG. (**d**). aB2GPI IgM. (**e**). Anti-Phosphatidyl serine/Prothrombin (aPS/PT) IgG. (**f**). aPS/PT IgM. (**g**). aB2GPI IgA. Left, sample1, Right, sample 2. The ordinate axis represents the level of antibodies in U / ml. Red line represent negative sera in the first sample that become positive in second sample. Dotted line represents the cutoff.

The presence of aPL in the second sample was significantly associated with the occurrence of thrombotic events (RR: 2.55, 95%CI: 1.38–4.74, *p* = 0.003). Dot and Line diagram of the paired samples for each aPL in patients with thrombotic events is shown in Figure 3.

When the three types of aPL were analyzed separately, thrombosis was significantly associated with the presence of classic aPL (RR: 3.59, 95%CI: 1.61–7.9, *p* = 0.003), anti-B2GPI IgA (RR: 2.09, 95%CI: 1.02–4.26, *p* = 0.047) and anti-PS/PT (RR: 3.36, 95%CI: 1.51–7.46, *p* = 0.005). The positivity of anti-SARS-CoV-2 antibodies in the first and second samples was not associated with thrombosis (Table 4A).

The appearance of thrombotic events after hospital admission occurred significantly earlier in aPL negative patients in the first sample (median 4 days, IQR: 3–7) than in aPL positive patients (median 17 days, IQR: 6–28, *p* = 0.016) (see Figure 2b). When aPL positivity in the second sample was considered, TE also occurred significantly earlier in aPL-negative patients (Figure 2c), median 4 days (IQR: 2.5–7), than in aPL positive (median 9 days, IQR: 6–25, *p* = 0.006). Analyzing the three types of aPL (classic aPL, IgA anti-B2GPI and anti-PS/PT), only IgA anti-B2GPI positive patients showed a significantly longer time to the onset of the first thrombotic event than the negative aPL patients (Supplementary Table S3).

The variables associated to TE with a *p*-value less than 0.11 were subjected to a multivariate analysis with a logistic regression model: presence of aPL in the first sample, age and hypertension (Table 4B). Presence of any aPL in the first sample (OR: 2.33, 95% CI: 1.03–5.29, *p* = 0.043) was identified as a significant and independent variable. (Table 4B). The multivariate analysis including aPL evaluation in the second sample more clearly showed that aPL positivity (OR: 3.71, 95% 1.71–8.05, *p* = 0.001) was a thrombosis-associated independent variable (Table 4B). The multivariate analysis using the three types of aPL independently (second sample, Supplementary Table S4) showed that only patients who were positive for IgA anti-B2GPI in the second sample had a significant increase in the risk of thrombosis (OR: 2.67, 95% (CI: 1.02–7, *p* = 0.046).

## 4. Discussion

In this study we have demonstrated that the presence of aPL in COVID-19 patients is similar to that observed in the general population of the same geographical area and that these antibodies persist after the appearance of the disease with very small variations. Additionally, we have observed that aPL positive patients have a higher incidence of thrombosis compared to aPL negative patients and that thrombotic events occurred significantly later in the first group.

The presence of aPL in APS is necessary but not sufficient to induce thrombosis formation. The concurrence of a “second hit” that involves activation of innate immunity and a proinflammatory microenvironment is necessary to trigger thrombotic episodes. This second hit could be severe infection, surgery, vascular procedures or trauma [37,38,39]. In patients with aPL, the immune response to SARS-CoV-2 infection acts as a second hit that increases the risk of triggering a thrombotic event, which would be added to the fact that the disease predisposes to thrombotic complications. The role of the inflammatory response as a second hit in the generation of thrombosis, is currently the subject of intense research in various fields, not only aPL related but also COVID-19 associated and cancer associated thrombosis [40,41].

Among the main inflammation-associated molecules that are proposed as activators of thrombosis, the High mobility group box-1 (HMGB-1) stands out. HMGB-1 is a damage-associated molecular pattern protein that, when is secreted by damaged cells, exerts a strongly inflammatory activity binding to receptors as receptor for advanced glycation end products (RAGE) and Toll-like receptors 3 and 4. The link between HMGB-1 and thrombosis appears to be focused on the interaction with platelets, NET and coagulation and fibrinolysis factors [42,43,44].

The fact that thrombotic events in aPL patients occurred later than in aPL negative ones suggests that the mechanism involved in these aPL positive patients may be different and additional to that directly related to the infection.

The presence of autoantibodies (antinuclear, aPL and anti-thyroids) is more frequent in the elderly than in young individuals [45,46]. Most of these antibodies are not part of an autoimmune response but are elaborated in response to an increase in apoptotic cells in the context of tissue damage (scavenger antibodies) from the senescence process [47]. Even though blood donors are a very homogeneous group of people in good health, those 65 years and older and people between 50 and 65 years old are underrepresented (most are between 30 and 50 years old) so the prevalence of autoantibodies in blood donors is lower than in the real population. To avoid the bias that using blood donors would entail, we have used a control population elaborated with an age distribution representative of the general population of the area from which the patients come. We have not observed significant differences in the prevalence of aPL when we compare with reference population but a significantly higher prevalence when compared to a group of blood donors (not matched by age). The higher prevalence of aPL reported in other studies with COVID-19 patients versus their own control population may be due to the lower mean age of the control populations with which they were compared, which evidently have a lower prevalence of autoantibodies. In the meta-analysis, carried out by Taha and Samavati, in which the prevalence of aPL in COVID-19 patients was assessed by evaluating 21 publications, all studies reported prevalence without a control group [22]. Therefore, it must be assumed that in these 21 studies, the comparisons were made using the aPL prevalence published in the literature, which are based on blood donor cohorts. In the few published studies that report a control population, most of them do not use age-matched control populations and when they use matched groups, these are very small [48].

The prevalence differences found in the literature are mainly due to the number of parameters analyzed and the methodology used. There are studies where only anti-CL and anti B2GPI IgG/M are compared, other groups also incorporate the IgA isotype and others include LA. Very few also incorporate anti-PS/PT.

The prevalence and the titer of aPL scarcely varied between the acute phase of the disease and post-convalesce, while the antibodies against SARS-CoV-2 antigens were strongly increased. These facts suggest that the presence of aPL is independent of infection in most patients with aPL. SARS-CoV-2 infection, in patients who carried aPL, hardly affects the presence of these antibodies.

In a minority of patients with aPL with a low titer close to the cutoff, SARS-CoV-2 infection has been a stimulus to increase the production of antibodies to place them above the cutoff point in the second sample. The SARS-CoV-2 infection had a dual role, main as a “second hit” and in some patients as a stimulus for the production of aPL. 

The fact that the association of aPL with thrombosis is significant only when we assess the post-event serum sample (second sample) does not imply any limitation since the presence of aPL is being assessed a posteriori. In the APS patients the determination of aPL is usually done after the occurrence of the thrombotic event. Except in cases of aPL-related thrombosis in SLE and after transplant surgery, it is extremely rare to find aPL evaluations prior to the second hit [49].

The classic aPL were the main subgroup of aPL significantly involved in COVID-19 associated thrombosis. Secondly, the association with anti-B2GPI of the IgA isotype also stands out. The detection of these IgA antibodies was not carried out with ALBIA (as was done with the classic aPL) but rather was done with a solid phase assay test because the tests based on antigen coated-beads have low sensitivity for this antibodies [50]. The studies that demonstrate a greater association of IgA anti-B2GPI presence with thrombosis in COVID patients used solid phase assays [51].

Some authors have proposed that the presence of aPL and thrombotic events in COVID-19 patients could represent a secondary form of APS [52,53]. The aPL associated with secondary APS caused by infections usually disappeared in a short period of time [7,43,44,45]. Xiao et al. propose that the dynamics of aPL in COVID-19 are similar to those in other infections [20]. By contrast, our results showed that the dynamics of aPL in COVID-19 patients are clearly different from that antibodies linked to the infection. Most of these patients are already positive at the beginning of the infection and continue to be so at least 12 weeks later. Our data suggest that the aPL-related COVID-19 thrombopathy is a primary APS which debuts in the context of a strong response to infection that acts as a second hit. In addition to this mechanism, in the acute phase of the disease, the production of thrombi would occur after a vascular dysfunction secondary to an imbalance in the molecules that regulate immune activity and tissue cleaning and repair [21,54,55].

Based on the data provided here, the presence of aPL in COVID-19 patients cannot continue to be considered as an epiphenomenon. Patients with aPL in our study who had thrombotic events after SARS-CoV-2 infection strictly comply with the Sidney criteria: presence of an event and positivity of aPL in two samples separated by 12 weeks. What remains to be determined is whether these patients can continue to suffer new thrombotic events in the face of future activations of the immune response that act as a second hit. Faced with this possibility, we suggest that, as has been done in Primary-APS patients, aPL positive patients should be kept under observation during a longer period as well as that 4 months of prophylaxis should be considered (as done in any other APS patient) [56] especially when d-dimer level was greater than 500 ng/mL [57]. More studies are needed about the necessity of prophylaxis.

The two main strengths of this work are the size of the cohort, one of the largest prospective cohorts that analyzes the effect of aPL on COVID-19 thrombosis, and that comparisons of prevalence and levels of aPL in patients with COVID -19 are performed in front of a healthy people group who have the same age structure as the general population, so they are more real and not as artificially high as when compared with blood donors. However, this work has several limitations. One of the main limitations is that it is a single-center study with a relatively small sample size. Although the size is sufficient to assess the influence of aPL in convalescence and determine the association of the presence of aPL with thrombotic events, further investigation is needed to assess the individual impact of different aPL on thrombosis risk. Therefore, the association of the presence of aPL with thrombotic events should be studied more carefully in subsequent studies with a greater number of patients.

The presence of LA was only evaluated in 67 patients. Although these patients only represent 20% of the cohort, this is a sufficient number to verify the prevalence and the lack of association with COVID-19 complications. Our results are in line with many studies that have highlighted the increased positivity rate in LA in COVID-19 patients [32,58,59] and have shown that many patients with COVID-19 have false positives of LA, related to the state of inflammation [26,60] and that these did not correlate with thrombotic risk [61].

## 5. Conclusions

Prevalence of aPL in COVID-19 patients is similar to the healthy population of the same age range. The majority of aPL carriers at the onset of the disease remain positive after more than 12 weeks of convalesce. Moreover, aPL in COVID-19 are associated with belated thrombotic risk. Thrombotic events in COVID-19 patients might be produced by at least two mechanisms: early thrombus mediated by thrombophilia and immune dysregulation inherent to tissue damage after infection and the belated thrombus mediated by the activation of a latent APS before the concurrence of a second hit, as is the strong immune response to infection. These conclusions should be validated by subsequent studies with a larger multicenter series.

## Figures and Tables

**Table 1:** Prevalence of antiphospholipid antibodies in reference population compared to COVID-19 patients (first and second serum samples). *p* values refer to the reference population.

Antibodies	Reference Population *n* = 143	COVID-19 Patients (*n* = 360)			
		First Sample	*p*-Value	Second Sample	*p*-Value
^1^ Any aPL	20 (14%)	63 (17.5%)	0.338	63 (17.5%)	0.338
^1^ Classic aPL	4 (2.8%)	16 (4.4%)	0.460	15 (4.2%)	0.608
^2^ Anti-B2GPI IgG	1 (0.7%)	4 (1.1%)	1.0	5 (1.4%)	1.0
^2^ Anti-B2GPI IgM	4 (2.8%)	10 (2.8%)	1.0	11 (3.1%)	1.0
^3^ Anti-CL IgG	2 (1.4%)	8 (2.2%)	0.732	12 (3.3%)	0.368
^3^ Anti-CL IgM	4 (2.8%)	10 (2.8%)	1.0	16 (4.4%)	0.460
^2^ Anti-B2GPI IgA	9 (6.3%)	40 (11.1%)	0.100	42 (11.7%)	0.072
^4^ Anti-PS/PT	7 (4.9%)	15 (4.2%)	0.719	16 (4.4%)	0.827
^4^ Anti-PS/PT IgG	4 (2.8%)	7 (1.9%)	0.517	8 (2.2%)	0.748
^4^ Anti-PS/PT IgM	3 (2.1%)	9 (2.5%)	1.0	9 (2.5%)	1.0

^1^ aPL, antiphospholipid antibody. ^2^ B2GPI, β2-glycoprotein-I. ^3^ CL, cardiolipin. ^4^ PS/PT, phosphatidylserine/prothrombin.

**Table 2:** Paired samples analysis (Wilcoxon test) of antiphospholipid and IgG anti-SARS-CoV-2 virus levels in serum of COVID-19 patients. Median difference index is calculated by dividing Hodges–Lehmann by the value of the median of the first sample.

	First Sample				Second Sample				Wicolxon Test		
Antibodies	Mean	SEM	Median	IQR	Mean	SEM	Median	IQR	Hodges–Lehmann Median Difference	*p*–Value	Median Difference Index
^1^ Anti-B2GPI IgG	3.1	14.7	1.4	(1.4–1.4)	3.1	14.6	1.4	(1.4–1.4)	0	0.396	0
^1^ Anti-B2GPI IgM	2.9	5.9	1.1	(0.4–2.6)	2.9	7.8	1	(0.4–2.3)	−0.1	0.153	−0.09
^2^ Anti-CL IgG	3.9	16.9	1.6	(1.6–1.6)	3.8	16.8	1.6	(1.6–1.6)	0	0.275	0
^2^ Anti-CL IgM	3.1	5.7	1.2	(0.5–2.7)	2.7	5.9	0.9	(0.3–2.3)	−0.1	0.019	−0.08
^1^ Anti-B2GPI IgA	10.5	20.4	4.4	(2.9–7.2)	10.6	21.1	4.4	(3.2–6.9)	0.13	0.440	0.03
^3^ Anti-PS/PT IgG	8	12.2	6	(5.2–7.4)	9	12.7	6.7	(5.8–8.1)	0.67	<0.001	0.11
^3^ Anti-PS/PT IgM	13.3	18.4	9.3	(6.5–14.3)	13.9	16.9	9.5	(7–14.9)	0.54	0.141	0.06
^4^ Anti-NCap-SARS-CoV2	34.2	38.6	14	(1–65.3)	70.4	37.3	95	(31.8–101)	39.5	<0.001	2.82
^5^ Anti-RBD-SARS-CoV2	37.2	41.1	15.5	(1–86)	91.8	26.7	101	(101–101)	50.3	<0.001	3.25
^6^ Anti-S1-SARS-CoV2	30.7	39.2	7.5	(0.6–70)	89.2	29.7	101	(101–101)	55	<0.001	7.33
^7^ Anti-S2-SARS-CoV2	13.3	22.7	4	(0.6–15)	30.8	26.5	23	(12–40)	15.2	<0.001	3.8

^1^ B2GPI, β2-glycoprotein-I. ^2^ CL, cardiolipin. ^3^ PS/PT, phosphatidylserine/prothrombin. ^4^ NCap, nucleocapsid. ^5^ RBD, receptor-binding domain. ^6^ S1, Spike 1. ^7^ S2, spike 2.

**Table 3:** Analysis of the agreement between the evaluations of aPL in the first and second samples. Strength of Agreement was rated according McHugh recommendations [36].

Antibodies	Positive Patients First Sample	Positive Patients Second Sample	Weighted Kappa	95% CI	Strength Agreement
^1^ Any aPL	63 (17.5%)	63 (17.5%)	0.75	(0.66–0.84)	Moderate
^1^ Any classic aPL	16 (4.4%)	15 (4.2%)	0.90	(0.79–1)	Strong
^2^ Anti-B2GPI IgG	4 (1.1%)	5 (1.4%)	0.89	(0.67–1)	Strong
^2^ Anti-B2GPI IgM	10 (2.8%)	11 (3.1%)	0.85	(0.69–1)	Strong
^3^ Anti-CL IgG	8 (2.2%)	12 (3.3%)	0.93	(0.80–1)	Almost Perfect
^3^ Anti-CL IgM	10 (2.8%)	16 (4.4%)	0.91	(0.78–1)	Almost Perfect
^2^ Anti-B2GPI IgA	40 (11.1%)	42 (11.7%)	0.92	(0.85–0.98)	Almost Perfect
^4^ Any anti-PS/PT	15 (4.2%)	16 (4.4%)	0.43	(0.20–0.65)	Weak
^4^ Anti-PS/PT IgG	7 (1.9%)	8 (2.2%)	0.52	(0.21–0.83)	Weak
^4^ Anti-PS/PT IgM	9 (2.5%)	9 (2.5%)	0.43	(0.14–0.72)	Weak

^1^ aPL, antiphospholipid antibody. ^2^ B2GPI, β2-glycoprotein-I. ^3^ CL, cardiolipin. ^4^ PS/PT, phosphatidylserine/prothrombin.

**Table 4:** Characteristics of the patients who developed thrombotic events during the first six months from the diagnosis of COVID-19. A. Univariate analysis of thrombosis associated factors. B. Multivariate analysis of factors associated to thrombosis events during follow-up (6 months).

Variables	Thrombosis (*n* = 37)	No Thrombosis (*n* = 323)	*p*-Value	Relative Risk	95% CI
**A. Thrombosis Associated Factors.**					
Sex (men)	24 (64.9%)	189 (58.5%)	0.457		
Age years (median and IQR)	54 (45.5–61.3)	60 (47.3–70.0)	0.050		
Diabetes	6 (16.2%)	31 (9.6%)	0.209		
Smoker	4 (10.8%)	69 (21.4%)	0.193		
Hypertension	7 (18.9%)	108 (33.4%)	0.073		
Obesity	13 (35.1.9%)	87 (27.9%)	0.292		
Treated at ICU	5 (13.5%)	32 (9.9%)	0.494		
Antibodies First sample					
^1^ Any aPL positive	10 (27%)	53 (16.4%)	0.107		
^1^ Classic aPL	5 (13.5%)	11 (3.4%)	0.005	3.36	1.51–7.46
^2^ Anti-B2GPI IgA	6 (16.2%)	34 (10.5%)	0.297		
^3^ Any anti-PS/PT	4 (10.8%)	11 (3.4%)	0.056		
Lupus anticoagulant (*n* = 67)	5 (16.7%)	8 (21.6%)	0.750		
Anti-SARS-CoV2 (*n* = 128)					
^4^ Anti-Ncap	7 (63.6%)	62 (53.9%)	0.536		
^5^ Anti-RBD	8 (72.7%)	62 (53.9%)	0.230		
^6^ Anti-S1	6 (54.5%)	53 (46.1%)	0.591		
^7^ Anti-S2	4 (36.4%)	40 (34.8%)	0.916		
Antibodies Second sample					
^1^ Any aPL positive	13 (35.1%)	50 (15.5%)	0.003	2.55	1.38–4.74
^1^ Classic aPL	5 (13.5%)	10 (3.1%)	0.003	3.59	1.61–7.9
^2^ Anti-B2GPI IgA	8 (21.6%)	34 (10.5%)	0.047	2.09	1.02–4.26
^3^ Any anti-PS/PT	5 (13.5%)	11 (3.4%)	0.005	3.36	1.51–7.46
Anti-SARS-CoV2 (*n* = 128)					
^4^ Anti-Ncap	7 (63.6%)	62 (53.9%)	0.536		
^5^ Anti-RBD	8 (72.7%)	62 (53.9%)	0.230		
^6^ Anti-S1	6 (54.5%)	53 (46.1%)	0.591		
^7^ Anti-S2	4 (36.4%)	40 (34.8%)	0.916		
**B. Logistic Regression Multivariate Analysis.**					
**Variable**	**Odds Ratio**	**95%CI**	***p*-Value**		
First serum sample					
^1^ Any aPL positive	2.33	1.03–5.29	0.043		
Hypertension	0.49	0.21–1.17	0.107		
Age years	0.98	0.96–1	0.067		
Second serum sample					
^1^ Any aPL positive	3.71	1.71–8.05	0.001		
Hypertension	0.45	0.19–1.08	0.075		
Age years	0.98	0.95–1	0.053		

^1^ aPL, antiphospholipid antibody. ^2^ B2GPI, β2-glycoprotein-I. ^3^ PS/PT, phosphatidylserine/prothrombin. ^4^ Ncap, nucleocapsid. ^5^ RBD, receptor-binding domain. ^6^ S1, Spike 1. ^7^ S2, spike 2.

## Data Availability

The raw data supporting the conclusions of this article will be made available by the authors, without undue reservation.

## Supplementary Materials

The following are available online at https://www.mdpi.com/article/10.3390/biomedicines9080899/s1, Figure S1: Algorithm of disposition and outcomes, Figure S2: Number and type of aPL positivity in the COVID-19 patients Table S1: Prevalence of antiphospholipid antibodies in a group of anonymous blood donors compared to reference population and COVID-19 patients. Table S2: Outputs during the follow-up in patients with antiphospholipid antibodies * calculated in comparison with the rest of the patients, Table S3: Time of appearance of the first thrombotic event (from hospital admission), depending on the presence of antiphospholipid antibodies, Table S4: Logistic regression multivariate analysis of thrombosis associated factors.
