# Supplementary material for: Anti-Phospholipid Antibodies and COVID-19 Thrombosis: A Co-Star, Not a Supporting Actor
Source: Biomedicines. 2021 Jul 27;9(8):899. doi: 10.3390/biomedicines9080899 (PMC8389622; doi:10.3390/biomedicines9080899)
Supplement: Supplementary file 1 [file biomedicines-09-00899-s001.zip › biomedicines-1272871-supplementary.pdf]

Supplementary Material

Table S1. Prevalence of antiphospholipid antibodies in a group of anonymous blood donors compared to reference population and COVID-19 patients at the time of admission to the hospital. aPL, antiphospholipid antibody. B2GPI, β2-glycoprotein-I. PS/PT, phosphatidylserine/prothrombin.

Antibodies	Blood Donors	Reference Population		COVID-19 Patients	
	**(*n* = 320)**	**(*n* = 143)**	***p*-value**	**(*n* = 360)**	***p*-value**
Any aPL	15 (4,7%)	20 (14%)	<0.001	63 (17.5%)	<0.001
Classic aPL	2 (0,6%)	4 (2.8%)	0.076	16 (4.4%)	0.002
Anti-B2GPI IgA	4 (1,3%)	9 (6.3%)	0.004	40 (11.1%)	<0.001
Anti-PS/PT	10 (3,1%)	7 (4.9%)	0.394	15 (4.2%)	0.471

Table S2. Outputs during the follow-up in patients with antiphospholipid antibodies * calculated in comparison with the rest of the patients. B2GPI, β2-glycoprotein-I, CL, cardiolipin. PS/PT, phosphatidylserine/prothrombin.

Antibodies	First Sample		*p*-value*	Second Sample		*p*-value*
			Patients with Thrombotic Events			
Any aPL	10	(15.9%)	0.107	13	(20.6%)	0.003
Classic aPL	5	(31.3%)	0.016	5	(33.3%)	0.012
Anti-B2GPI IgA	6	(15%)	0.297	8	(19%)	0.046
Anti-PS/PT	4	(26.7%)	0.056	5	(31.3%)	0.016
			Patients with Ventilatory Failure			
Any aPL	13	(20.6%)	0.214	12	(19%)	0.120
Classic aPL	2	(12.5%)	0.253	1	(6.7%)	0.093
Anti-B2GPI IgA	9	(22.5%)	0.502	8	(19%)	0.220
Anti-PS/PT	3	(20%)	0.768	3	(18.8%)	0.572
			Patients Treated in ICU			
Any aPL	5	(7.9%)	0.649	6	(9.5%)	0.828
Classic aPL	1	(6.3%)	1	1	(6.7%)	1
Anti-B2GPI IgA	4	(10%)	1	4	(9.5%)	1
Anti-PS/PT	0	(0%)	0.382	1	(6.3%)	1

Table S3. Time of appearance of the first thrombotic event (from hospital admission), depending on the presence of antiphospholipid antibodies. B2GPI, β2-glycoprotein-I, CL, cardiolipin. PS/PT, phosphatidylserine/prothrombin. * Median difference, p-values and Median difference index were calculated in comparison with aPL negative patients.

Antibodies	Mean (Days)	Median (Days)	IQR	Hodges-Lehmann Median Difference*	*p*-value*	Median Difference Index*
aPL negative	6.3	4	(2.5–7)	-	-	-
Any aPL positive	29.1	9	(6–25)	5	0.006	1.25
Classic aPL	37.2	6	(4–51)	-	0.309	-
Anti-B2GPI IgA	30	12.5	(7–21)	6	0.024	1.5
Anti-PS/PT	57.2	28	(4.8–109)	-	0.092	-

Table S4. Logistic regression multivariate analysis of thrombosis associated factors. The variable APL-positivity has been divided into the three types of aPL: classic aPL, IgA anti-B2GPI and Anti-PS/ PT.

Variable	Odds Ratio	95%CI	*p*-value
	First Serum Sample		
Classic aPL	3.98	1.19–3.35	0.025
Anti-B2GPI IgA	1.68	0.58–4.84	0.341
Any anti-PS/PT	2.77	0.75–0.26	0.127
Hypertension	0.51	0.21–1.22	0.13
Age years	0.98	0.96–1	0.07
	Second Serum Sample		
Classic aPL	3.65	1–3.29	0.049
Anti-B2GPI IgA	2.67	1.02–7	0.046
Any anti-PS/PT	3.16	0.9–1.08	0.072
Hypertension	0.46	0.19–1.12	0.087
Age years	0.98	0.96–1	0.082

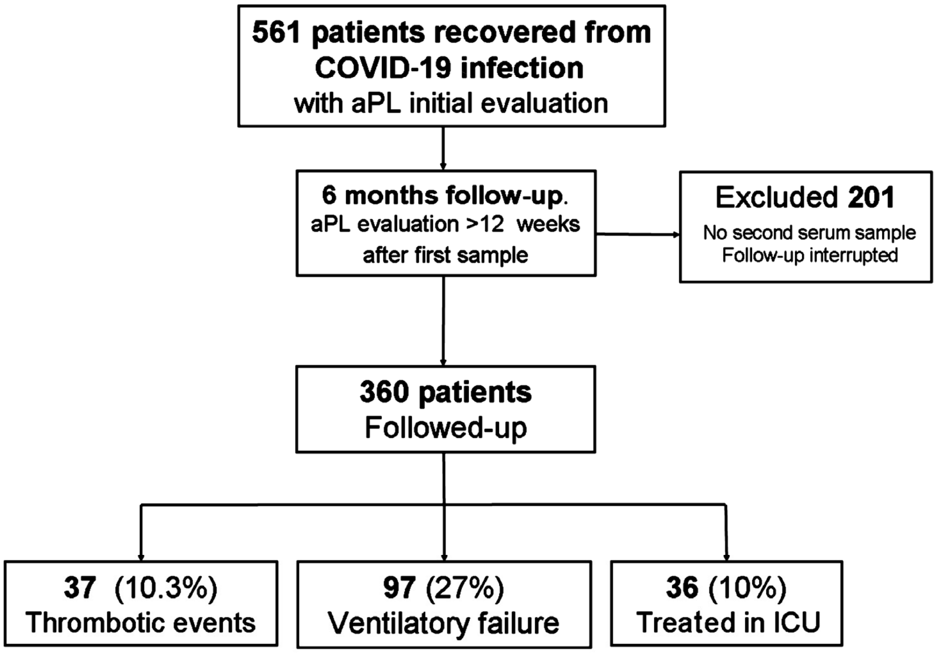

Figure S1. Algorithm of disposition and outcomes.

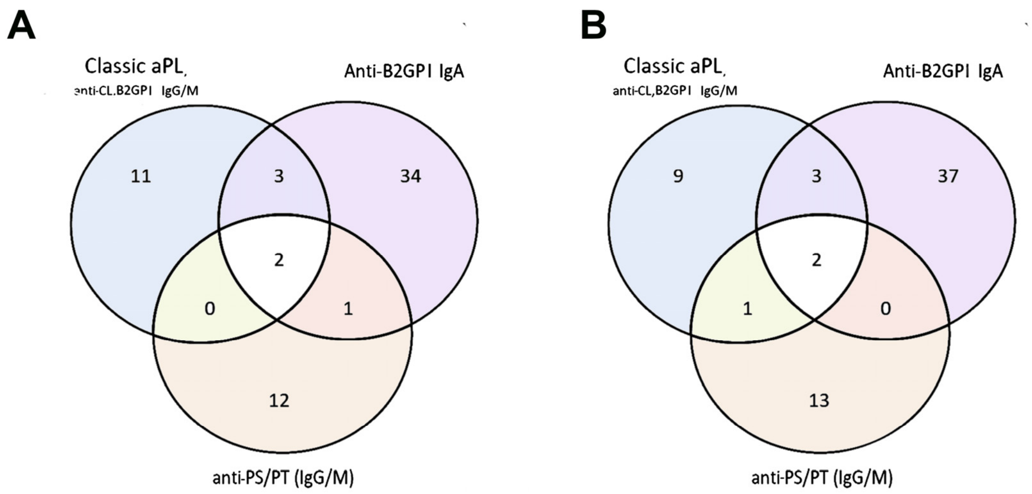

Figure S2. Number and type of aPL positivity in the COVID-19 patients. A. First serum sample. B. Second serum sample. B2GPI, β2-glycoprotein-I, CL, cardiolipin. PS/PT, phosphatidylserine/prothrombin.
